# Parameter-efficient fine-tuning for low-resource text classification: a comparative study of LoRA, IA^3^, and ReFT

**DOI:** 10.3389/fdata.2025.1677331

**Published:** 2025-12-02

**Authors:** Steve Nwaiwu

**Affiliations:** Data and Information Science, Faculty of Science and Technology, Rajamangala University of Technology, Pathum Thani, Thailand

**Keywords:** machine learning, deep learning, single modal, multi-modal learning, text processing

## Abstract

The successful application of large-scale transformer models in Natural Language Processing (NLP) is often hindered by the substantial computational cost and data requirements of full fine-tuning. This challenge is particularly acute in low-resource settings, where standard fine-tuning can lead to catastrophic overfitting and model collapse. To address this, Parameter-Efficient Fine-Tuning (PEFT) methods have emerged as a promising solution. However, a direct comparative analysis of their trade-offs under unified low-resource conditions is lacking. This study provides a rigorous empirical evaluation of three prominent PEFT methods: Low-Rank Adaptation (LoRA), Infused Adapter by Inhibiting and Amplifying Inner Activations (IA^3^), and a Representation Fine-Tuning (ReFT) strategy. Using a DistilBERT base model on low-resource versions of the AG News and Amazon Reviews datasets, the present work compares these methods against a full fine-tuning baseline across accuracy, F1 score, trainable parameters, and GPU memory usage. The findings reveal that while all PEFT methods dramatically outperform the baseline, LoRA consistently achieves the highest F1 scores (0.909 on Amazon Reviews). Critically, ReFT delivers nearly identical performance (~98% of LoRA's F1 score) while training only ~3% of the parameters, establishing it as the most efficient method. This research demonstrates that PEFT is not merely an efficiency optimization, but a necessary tool for robust generalization in data-scarce environments, providing practitioners with a clear guide to navigate the performance—efficiency trade-off. By unifying these evaluations under controlled conditions, this study advances beyond fragmented prior research and offers a systematic framework for selecting PEFT strategies.

## 1 Introduction

The advent of large-scale, pre-trained transformer models (Vaswani et al., 2017) has fundamentally reshaped the Natural Language Processing (NLP) landscape. Architectures like BERT (Devlin et al., 2019) and its successors have become the backbone of a vast array of downstream tasks, achieving state-of-the-art performance by leveraging the knowledge encoded in billions of parameters. The standard paradigm for adapting these powerful models to specific applications is full fine-tuning, where all of the model's weights are updated on a task-specific dataset.

However, the efficacy of full fine-tuning is predicated on access to substantial computational resources and large, high-quality datasets. In many real-world scenarios, particularly in low-resource environments, these prerequisites are not met. When faced with limited data, the immense capacity of large models makes them prone to severe overfitting. This can lead to a catastrophic failure to generalize, where the model memorizes the training data's noise rather than learning the underlying task, resulting in performance no better than random chance. This challenge highlights a critical bottleneck that limits the widespread and democratic application of large language models (Zhang et al., 2025).

To mitigate these issues, the research community has developed a suite of Parameter-Efficient Fine-Tuning (PEFT) techniques. These methods aim to adapt pre-trained models by updating only a small fraction of their parameters, drastically reducing computational and storage costs while preserving the rich knowledge of the base model (Wu et al., 2024). PEFT strategies are diverse, ranging from adding small, trainable “adapter” modules between layers (Houlsby et al., 2019), to reparameterizing weight matrices with low-rank updates like LoRA (Hu et al., 2021), to selectively unfreezing and updating only specific components of the model, such as biases or normalization layers, in a strategy referred to as Representation Fine-Tuning (ReFT).

While these methods have been individually validated, most existing studies examine them in isolation or on narrow task settings. As a result, practitioners lack a unified data-driven guide to choose between PEFT strategies under consistent low-resource conditions (Lialin et al., 2024). Addressing this gap requires a systematic comparison that quantifies both performance and efficiency under controlled conditions, thereby clarifying the trade-offs inherent in different PEFT designs.

Research Question. The central question addressed in this study is: *How do distinct PEFT strategies—LoRA, IA*^3^ *(Liu et al.*, *2022**), and ReFT—compare in balancing task performance and computational efficiency for low-resource text classification?* By answering this question, the study aims to advance the field beyond fragmented evaluations and provide clear empirical guidance for practitioners.

The contributions of this research are threefold:

- A systematic, head-to-head comparison of LoRA, IA^3^, and ReFT against a full fine-tuning baseline is conducted, measuring accuracy, F1 score, trainable parameter count, and GPU memory usage under unified low-resource conditions.
- The results demonstrate that under data-scarce scenarios, PEFT is not merely an efficiency optimization but a necessary condition for achieving robust model performance and avoiding catastrophic overfitting.
- A performance–efficiency frontier is established, offering practitioners an actionable framework for selecting the most appropriate PEFT strategy according to their deployment constraints.

This paper is structured as follows. Section 2 reviews related work on parameter-efficient fine-tuning. Section 3 details the experimental methodology, including datasets, models, and evaluation metrics. Section 4 presents and analyzes the results of the experiments. Finally, Section 5 discusses the implications of the findings and concludes the article with directions for future work.

## 2 Related work

The challenge of adapting large pre-trained language models (PLMs) without incurring prohibitive computational costs has spurred the development of a diverse field of research in Parameter-Efficient Fine-Tuning (PEFT). The core principle of PEFT is to freeze the vast majority of a PLM's parameters and update only a small, strategically chosen subset. This approach not only drastically reduces memory and storage requirements, but also often acts as a powerful regularizer, preventing catastrophic forgetting (Wang et al., 2025) and overfitting commonly observed during full fine-tuning of limited data. The landscape of PEFT methods can be broadly categorized into three main approaches: additive, selective, and reparameterization-based methods.

Additive methods, one of the most popular strategies, involve augmenting the pre-trained model with new, trainable modules or parameters. The seminal work in this area introduced “adapters,” which are small feedforward networks inserted into the bottleneck between the layers of a transformer. During fine-tuning, only the parameters of these lightweight adapters are trained. This approach has been shown to achieve performance comparable to full fine-tuning while updating only a fraction of a percent of the total parameters. More recent additive methods have sought even greater efficiency. IA^3^ (Infused Adapter by Inhibiting and Amplifying Inner Activations) simplifies this further by learning just three rescaling vectors per transformer block to amplify or suppress activations, achieving strong performance with a minimal parameter budget (Ansell et al., 2024; Sehanobish et al., 2024).

Reparameterization methods modify the fine-tuning process by transforming the weight update matrices. The most prominent example is Low-Rank Adaptation, or LoRA. Instead of directly updating a large weight matrix *W*, LoRA represents the update as a low-rank decomposition, Δ*W* = *BA*, where *B* and *A* are two much smaller matrices. Only *B* and *A* are trained, significantly reducing the number of trainable parameters. A key advantage of LoRA is that, after training, the update Δ*W* can be merged back into the original weight *W*, which means that it does not introduce additional inference latency. Due to its effectiveness and simplicity, LoRA has become a de facto standard for efficient tuning (Bershatsky et al., 2024).

Selective methods take a different approach, forgoing the addition of new parameters and instead carefully choosing a small subset of the model's original parameters to update. This is one of the simplest forms of PEFT, with early strategies involving heuristic choices like unfreezing only the top layers of a network. A more principled variant is BitFit (Ben Zaken et al., 2022), which proposes fine-tuning only the bias parameters of the model. The present study evaluates a similar strategy termed Representation Fine-Tuning (ReFT), which focuses on updating only the parameters of the final classification head and the layer normalization modules throughout the network. These parameters are critical for adapting the model's representations to a new task's distribution without disturbing the core knowledge stored in the main weight matrices (Wu et al., 2024).

While these PEFT methods have been extensively validated, much of the existing research evaluates them in isolation or compares only a narrow subset (e.g., LoRA vs. IA^3^ on code generation tasks, as in recent studies). A clear and direct comparison between these different families of methods (additive, reparameterization, and selective) under unified, controlled low-resource conditions for common NLP tasks is less established (Hadji-Kyriacou and Arandjelovic, 2023). Recent surveys and benchmarks (Lialin et al., 2024; Wang et al., 2025; Zhang et al., 2025) further highlight that systematic evaluations are needed to guide practitioners, while emerging hybrid methods (Qi et al., 2025) suggest new directions, but also underscore the lack of consistent baselines. By situating LoRA, IA^3^, and ReFT side-by-side under standardized conditions, this research addresses this gap and provides an empirical map of the performance–efficiency trade-offs that existing fragmented studies leave unresolved.

## 3 Methodology

To provide a rigorous and controlled comparison of the selected fine-tuning strategies, an experimental pipeline was designed to standardize the base model, datasets, and training hyperparameters across all conditions. This ensures that any observed differences in performance and efficiency can be directly attributed to the fine-tuning method itself.

### 3.1 Datasets and preprocessing

Two well-established public datasets for text classification were selected, simulating low-resource conditions through aggressive subsampling. These datasets were chosen because they are widely used benchmarks in the PEFT and low-resource learning literature, enabling both comparability with previous studies and replicability of the results. In addition, they represent two distinct classification scenarios, multiclass topic categorization and binary sentiment analysis, ensuring diversity in task structure.

AG News: A 4-class topic classification dataset (Li, 2024). The classes are *World, Sports, Business*, and *Sci/Tech*. From the original training set, a low-resource sample was created by randomly selecting 250 examples per class (balanced), resulting in a training set of 1,000 examples. The full official test split was used for evaluation. Inputs consist solely of text: the provided *Title* and *Description* fields were concatenated into a single string (with a delimiter), and no handcrafted or auxiliary features were used. The outputs are integer class labels in {0, 1, 2, 3} obtained by mapping the original indices of the dataset to zero-based labels.

*Example entries:*

- *Title:* “Apple launches new AI-powered chip”—*Description:* “The company unveiled a processor designed for next-generation devices.” → *Label: 3 (Sci/Tech)*.
- *Title:* “Stock markets rally amid global optimism”—*Description:* “Major indexes rose as investors reacted positively to new trade data.” → *Label: 2 (Business)*.

All inputs are tokenized with distilbert-base-uncased using padding=max_length, truncation=True, and max_length=128, yielding fixed-length sequences of 128 tokens.

Amazon Polarity: A binary sentiment classification dataset (Zhang et al., 2015). The classes are *positive* and *negative*. A total of 1,000 training examples were randomly sampled from the original training split and evaluated on the full official test split. Inputs are text only: the *title* and *review text* fields were concatenated into a single string. The outputs are integer labels in {0, 1} produced by mapping the original polarity values of the dataset {1, 2} to zero-based classes.

*Example entries:*

- *Title:* “Great sound quality!”—*Review:* “These headphones exceeded expectations for clarity and comfort.” → *Label: 1 (Positive)*.
- *Title:* “Poor battery life”—*Review:* “Stopped working after two weeks and would not hold a charge.” → *Label: 0 (Negative)*.

All inputs are tokenized with distilbert-base-uncased using padding=max_length, truncation=True, and max_length=128, yielding fixed-length sequences of 128 tokens.

For both datasets, preprocessing involved combining the title and text (or description) fields into a single input string to provide maximum context to the model. The class labels were converted to a zero-indexed integer format (0–3 for AG News, 0–1 for Amazon Reviews). A fixed random seed of 42 was used for all sampling and label mappings to ensure strict reproducibility across runs.

### 3.2 Model and fine-tuning configurations

All experiments were carried out using the distilbert-base-uncased (Sanh et al., 2019) as the base model. DistilBERT was selected because it offers a strong balance between performance and computational efficiency: it retains approximately 97% of BERT's performance while being 40% smaller and 60% faster. This makes it an ideal testbed for controlled PEFT comparisons, allowing isolation of efficiency–performance trade-offs without the prohibitive costs of very large models (e.g., Llama-3 or Flan-T5).

Four distinct fine-tuning conditions were implemented. The PEFT methods were selected because they represent complementary paradigms within the broader landscape: reparameterization (LoRA), activation rescaling (IA^3^), and selective tuning (ReFT). Together, they provide a representative spectrum of design philosophies that currently shape PEFT research.

- Full Fine-Tuning (Baseline): In this standard approach, all parameters of the DistilBERT model (approximately 67 million) were unfrozen and updated during training.
- LoRA (Low-Rank Adaptation): Following the methodology of (Hu et al., 2021), trainable low-rank matrices were injected into the query (q_lin) and value (v_lin) layers of the self-attention mechanism. A rank (*r*) of 8 and a scaling factor (α) of 16 were selected. A rank of 8 is a standard value used in the literature that is widely recognized for providing a robust balance between representational capacity and parameter efficiency. All other pre-trained weights remained frozen.
- IA^3^ (Infused Adapter by Inhibiting and Amplifying Inner Activations): Based on the work (Liu et al., 2022), this method introduces learned vectors to rescale the key, value, and feed-forward network activations. This approach is even more parameter-efficient than LoRA. The original model weights were kept frozen.
- ReFT (Representation Fine-Tuning): This implementation of a selective tuning strategy froze the entire model except for the randomly initialized classification head and all LayerNorm parameters throughout the transformer blocks. This approach tests the hypothesis that adapting only the normalization statistics and the final output layer is sufficient for effective domain transfer.

### 3.3 Experimental setup and evaluation

To ensure a fair comparison, all models were trained using an identical set of hyperparameters. The AdamW optimizer was employed with a learning rate of 5e-4, a batch size of 16, and training for 5 epochs. While individual tuning could potentially yield further performance gains for each method, a fixed hyperparameter set was used to isolate the architectural impact of each PEFT strategy.

The performance and efficiency of the model were assessed using the following metrics.

- Performance Metrics: Accuracy and Macro F1 score (see Appendix 1 for formal definitions).
- Efficiency Metrics: Trainable Parameter Count and Peak GPU Memory Usage.

All experiments were performed within a Google Colab environment using a single NVIDIA T4 GPU. The implementation relied on the Hugging Face transformers and peft libraries.

## 4 Results

The experiments yielded clear and consistent results in both datasets, revealing significant differences in performance and efficiency among the four fine-tuning strategies. The complete findings are summarized in Table 1, which consolidates the performance and efficiency results for all methods.

**Table 1:** Consolidated performance and efficiency metrics for all fine-tuning methods across both low-resource datasets.

**Method**	**Dataset**	**Accuracy**	**F1 Score**	**Trainable params**	**Peak GPU (MB)**
Full fine-tuning	Amazon reviews	0.5000	0.3333	66,955,010	1431.91
IA^3^	Amazon reviews	0.8737	0.8737	601,346	773.81
ReFT	Amazon reviews	0.8890	0.8890	19,970	668.71
LoRA	Amazon reviews	0.9092	**0.9092**	739,586	796.12
Full fine-tuning	AG news	0.8443	0.8436	66,956,548	1431.94
IA^3^	AG News	0.8813	0.8808	602,884	773.84
ReFT	AG News	0.8814	0.8813	21,508	668.74
LoRA	AG News	0.8911	**0.8910**	741,124	903.64

Best F1 Score for each dataset is highlighted in bold.

### 4.1 Overall performance and efficiency

Table 1 presents the consolidated results from all eight experimental runs, detailing the final F1 score, accuracy, trainable parameter count, and peak GPU memory usage for each method on both the Amazon Reviews and AG News datasets.

#### 4.1.1 Detailed interpretation of Table 1

In both data sets, LoRA consistently achieves the highest F1 scores: 0.909 in Amazon Reviews and 0.891 in AG News, which represent absolute gains of +0.036 and +0.010 over IA^3^, respectively. ReFT achieves nearly identical F1 scores, within 1%–2% of LoRA, while requiring only about 3% of LoRA's trainable parameters (19,970 vs. 739,586 on Amazon) and consuming approximately 15%–20% less GPU memory. IA^3^, though more parameter efficient than LoRA, records slightly lower F1 performance and uses roughly 12% more GPU memory than ReFT, indicating less favorable scaling under tight resource budgets. The pattern is consistent across both datasets: LoRA maximizes performance, ReFT maximizes efficiency, and IA^3^ balances the two, but does not dominate either frontier. These trends reveal a clear efficiency hierarchy and confirm that PEFT techniques offer robust alternatives to full fine-tuning, which collapses under low-resource conditions.

### 4.2 Key observations

From the data, several key patterns emerge:

- Catastrophic failure of full fine-tuning: On the Amazon Reviews dataset, the full fine-tuning baseline failed to learn effectively, achieving an F1 score of 0.3333, which is indicative of model collapse and not better than random guessing for a binary task. On the AG News dataset, its performance was substantially lower than all PEFT methods.
- Superiority of PEFT methods: The three PEFT methods, LoRA, IA^3^, and ReFT, dramatically outperformed the full fine-tuning baseline in both datasets. On Amazon Reviews, the performance gap was particularly stark, with LoRA achieving an F1 score nearly 2.7 times higher than the baseline.
- LoRA as the top performer: In terms of absolute performance, LoRA consistently yielded the highest F1 score in both datasets, achieving 0.9092 on Amazon Reviews and 0.8910 on AG News.
- Efficiency of ReFT: The ReFT strategy achieved performance remarkably close to LoRA, reaching approximately 98% of LoRA's F1 score on both datasets. It accomplished this while training a minute fraction of the parameters—only 19,970 for Amazon Reviews, compared to LoRA's 739,586. This represents a 97.3% reduction in trainable parameters for a minimal performance trade-off. ReFT also consistently recorded the lowest peak GPU memory usage.

## 5 Discussion

The results of this study provide a clear and compelling narrative about the role of fine-tuning strategies in low-resource contexts. The findings not only quantify the performance–efficiency trade-offs of various PEFT methods but also highlight a critical insight: parameter-efficient tuning is not merely an optimization for resource-constrained environments, but a fundamental requirement for achieving robust model generalization when data are scarce.

### 5.1 The overfitting catastrophe of full fine-tuning

The most striking result of the experiments is the catastrophic failure of the full fine-tuning baseline on the Amazon Reviews dataset. This outcome is a classic example of severe overfitting. With approximately 67 million parameters to update and only 1,000 training examples, the model possesses far too much capacity. Instead of learning the underlying patterns of sentiment, it effectively memorizes the noise and idiosyncrasies of the small training set. This finding serves as a crucial empirical validation that for low-resource NLP, the standard fine-tuning paradigm can be actively detrimental.

### 5.2 PEFT as a necessary regularizer

In stark contrast, all three PEFT methods achieved strong performance. By freezing the vast majority of the pre-trained weights, these methods act as a powerful form of regularization. They constrain the model's learning to a low-dimensional subspace, forcing it to adapt its existing robust representations rather than learning new ones from scratch on a small and unreliable dataset.

### 5.3 Analyzing the performance–efficiency frontier

While all PEFT methods were successful, they occupy different points on the performance–efficiency spectrum.

#### 5.3.1 LoRA: the performance leader

LoRA's consistent position as the top performer suggests that its strategy of applying low-rank updates to the self-attention mechanism is highly effective for task adaptation, modifying the most critical component for contextual understanding.

#### 5.3.2 ReFT: the efficiency champion and the power of recalibration

The success of the ReFT implementation is particularly noteworthy. Its remarkable performance, despite updating only ~20k parameters, can be attributed to the strategic importance of its target modules. LayerNorm parameters directly control the mean and variance of activations that flow through the network. By tuning only these, ReFT performs a highly targeted “distributional recalibration,” effectively adapting the model's internal representations to the statistical properties of the new task's data. This allows the model to be re-centered for a new domain without disturbing the complex, high-dimensional knowledge stored in the main weight matrices. This “less is more” approach demonstrates that surgical updates can be almost as effective as more extensive ones, making it an ideal choice for environments with extreme hardware constraints.

#### 5.3.3 IA^3^: an alternative approach

IA^3^ performed commendably but fell slightly behind LoRA and ReFT. The results may suggest that, for these specific classification tasks, directly modifying weight representations (LoRA) or normalization statistics (ReFT) provides a more direct path to high performance than rescaling activations alone.

### 5.4 Implications for practitioners

The findings provide a clear, data-driven framework. When absolute maximum performance is the only goal, LoRA is the recommended approach. However, when efficiency is a primary concern, ReFT offers an outstanding trade-off, delivering near-state-of-the-art performance for a fraction of the parameter and memory cost.

In deployment contexts, these trade-offs map directly to organizational constraints. For example, a startup with only a single GPU may find ReFT attractive, as it minimizes memory footprint while maintaining competitive performance. Conversely, a research laboratory with access to multi-GPU clusters may prefer LoRA to maximize accuracy, even at higher computational cost. In latency-critical pipelines, such as real-time content moderation, the reduced overhead of ReFT may outweigh small accuracy differences, whereas LoRA may be preferable in high-stakes applications such as biomedical or clinical text classification.

### 5.5 Advancing the field

This research advances the field of parameter-efficient fine-tuning by moving beyond fragmented evaluations. Prior studies have typically examined LoRA, IA^3^, or selective approaches in isolation, often under varying conditions that complicate comparison. By conducting a head-to-head analysis of three complementary paradigms under unified low-resource settings, this work provides a clear empirical map of the performance–efficiency frontier. This systematic perspective not only validates the necessity of PEFT in low-resource NLP but also establishes actionable guidelines for model selection, thereby filling a critical gap in both academic research and practical deployment.

## 6 Conclusion and future work

This study conducted a rigorous, head-to-head empirical comparison of three distinct Parameter-Efficient Fine-Tuning (PEFT) strategies: LoRA, IA^3^, and ReFT—against a traditional full fine-tuning baseline in a controlled, low-resource text classification setting. The findings lead to a clear and actionable conclusion: in data-scarce environments, the adoption of PEFT is not merely an efficiency choice, but a prerequisite for achieving robust model generalization.

The experiments demonstrated that attempting to fine-tune all parameters of a large pre-trained model on a small dataset can lead to catastrophic model failure, resulting in performance no better than random chance. In contrast, all evaluated PEFT methods successfully adapted the base model, yielding significant performance gains. Among them, LoRA consistently delivered the highest accuracy and F1 scores, establishing it as the premier choice for maximizing performance. Simultaneously, the ReFT implementation emerged as the champion of efficiency, achieving approximately 98% of LoRA's performance while requiring only 3% of the trainable parameters and consuming the least GPU memory.

The primary contribution of this research is the clear articulation of the performance–efficiency frontier, providing practitioners with an empirical framework to select the most appropriate tuning strategy for specific computational and deployment constraints.

### 6.1 Limitations

There are several limitations to acknowledge in this study. The analysis was confined to a single base model architecture, distilbert-base-uncased. While effective for a controlled comparison, these findings may not generalize perfectly to larger or more recent architectures (e.g., Llama-3, Flan-T5, or Mistral), which may exhibit different performance–efficiency dynamics.

Although the present analysis centers on two representative text classification benchmarks, these tasks were deliberately selected to capture both a multi-class topic categorization scenario (AG News) and a binary sentiment classification scenario (Amazon Reviews). This design ensures coverage of distinct label structures and linguistic characteristics within a limited experimental budget. Nevertheless, it does not encompass the full diversity of NLP task types. Other task families, such as natural language inference, question-answering, or summarization, may exhibit different adaptation behaviors and performance–efficiency patterns.

Finally, for the sake of a controlled comparison, a single fixed set of hyperparameters was employed across all methods. While this choice isolates architectural differences, prior studies have shown that PEFT methods can be sensitive to hyperparameter choices (e.g., rank, learning rate, and dropout). The reported results should therefore be interpreted as conservative baseline comparisons rather than method-specific performance ceilings.

### 6.2 Future work

The results from this study open up several compelling avenues for future research.

- Scaling up: An immediate next step is to replicate this comparative analysis on larger-scale models (e.g., Llama-3, Mistral, or Flan-T5) to investigate whether the observed performance–efficiency trade-offs hold as model size increases.
- Task diversification: The study should be expanded to include a wider range of NLP tasks, such as token classification, natural language inference, question answering, and text generation, to determine whether the optimal PEFT strategy is task dependent and whether the current findings generalize across task families.
- Hybrid methods: Future research could explore hybrid PEFT approaches, such as combining the structural updates of LoRA with the normalization tuning of ReFT (Qi et al., 2025), to determine whether a synergistic effect can push the performance–efficiency frontier even further.
- Data scaling analysis: A granular analysis of how the performance of each PEFT method changes as the number of training samples increases (e.g., from 100 to 5,000 examples) would provide deeper insight into their scaling properties and at what point full fine-tuning becomes a viable strategy.

In conclusion, this research confirms the indispensable role of PEFT in modern NLP and provides a clear and practical guide for navigating the available strategies within this rapidly evolving field.

## Data Availability

The original contributions presented in the study are included in the article/supplementary material, further inquiries can be directed to the corresponding author.

## Appendix

### Metric definitions

This study reports two primary evaluation metrics Accuracy and Macro-F1 computed on hard predictions ${ŷ}_{i}=\arg\underset{c}{\max}{\text{logits}}_{i,c}$, consistent with the evaluation code.

#### Accuracy

$$\begin{array}{l} \text{Accuracy}=\frac{TP+TN}{TP+TN+FP+FN}=\frac{1}{N}\overset{N}{\underset{i=1}{\sum }}\text{1}\left\{{ŷ}_{i}=y_{i}\right\}. \end{array}$$
(1)

#### Macro-F1

Let *K* be the total number of classes. For each class *k*∈{1, …, *K*}, define:

$$\begin{array}{l} {\text{Precision}}_{k}=\frac{TP_{k}}{TP_{k}+FP_{k}},\;{\text{Recall}}_{k}=\frac{TP_{k}}{TP_{k}+FN_{k}},\\ {\text{ F1}}_{k}=\frac{2{\text{Precision}}_{k}\cdot {\text{Recall}}_{k}}{{\text{Precision}}_{k}+{\text{Recall}}_{k}}. \end{array}$$

Then the overall Macro-F1 is given by

$$\begin{array}{l} \text{Macro-F1}=\frac{1}{K}\overset{K}{\underset{k=1}{\sum }}{\text{F1}}_{k}. \end{array}$$
(2)

#### Term definitions

Here, TP, TN, FP, and FN denote the numbers of *true positives, true negatives, false positives*, and *false negatives*, respectively. *N* is the total number of test samples. ŷ_*i*_ and *y*_*i*_ represent the predicted and gold (ground-truth) labels for sample *i*. **1**{·} is the indicator function, returning 1 if the condition inside is true and 0 otherwise. *K* denotes the total number of classes, and *k* indexes each class. Subscripts such as *TP*_*k*_, *FP*_*k*_, and *FN*_*k*_ refer to the per-class counts used in computing class-specific precision and recall.

#### Notes

(i) For binary classification (Amazon Polarity), Macro-F1 is the unweighted mean of the positive and negative class F1 scores (it is *not* the “positive-class F1”). (ii) In scikit-learn, classes with no predicted positives yield Precision_*k*_ = 0 and consequently F1_*k*_ = 0; this behavior is preserved in all reported results. (iii) Model selection throughout this study uses validation-set Macro-F1 as the criterion.

## References

[B1] Ansell A. Vulić I. Sterz H. Korhonen A. Ponti E. M. (2024). Scaling sparse fine-tuning to large language models. arXiv preprint, arXiv:2401.16405.

[B2] Ben Zaken E. Goldberg Y. Ravfogel S. (2022). “Bitfit: simple parameter-efficient fine-tuning for transformer-based masked language-models,” in Proceedings of the 60th Annual Meeting of the Association for Computational Linguistics (Volume 2: Short Papers), eds. S. Muresan, P. Nakov, and A. Villavicencio (Dublin, Ireland: Association for Computational Linguistics), 1–9. doi: 10.18653/v1/2022.acl-short.1

[B3] Bershatsky D. Cherniuk D. Daulbaev T. Mikhalev A. Oseledets I. (2024). Lotr: Low tensor rank weight adaptation. arXiv preprint, arXiv:2402.01376.

[B4] Devlin J. Chang M.-W. Lee K. Toutanova K. (2019). “Bert: pre-training of deep bidirectional transformers for language understanding,” in Proceedings of the 2019 Conference of the North American Chapter of the Association for Computational Linguistics: Human Language Technologies, Volume 1 (Long and Short Papers), eds. J. Burstein, C. Doran, and T. Solorio (Minneapolis, Minnesota: Association for Computational Linguistics), 4171–4186.

[B5] Hadji-Kyriacou A. A. Arandjelovic O. (2023). Context-peft: efficient multi-modal, multi-task fine-tuning. arXiv preprint, arXiv:2312.08900.

[B6] Houlsby N. Giurgiu A. Jastrzebski S. Morrone B. De Laroussilhe Q. Gesmundo A. . (2019). “Parameter-efficient transfer learning for NLP,” in Proceedings of the 36th International Conference on Machine Learning, eds. K. Chaudhuri, and R. Salakhutdinov (Long Beach, California, USA: PMLR), 2790–2799.

[B7] Hu E. J. Shen Y. Wallis P. Allen-Zhu Z. Li Y. Wang S. . (2021). Lora: Low-rank adaptation of large language models. arXiv preprint, arXiv:2106.09685.

[B8] Li X. (2024). Ag News and IMDB. Dataset. Includes AG News and IMDB benchmarks.

[B9] Lialin V. Deshpande V. Yao X. Rumshisky A. (2024). Scaling down to scale up: a guide to parameter-efficient fine-tuning. arXiv preprint, arXiv:2303.15647v152.

[B10] Liu H. Tam D. Muqeeth M. Mohta J. Huang T. Bansal M. . (2022). “Few-shot parameter-efficient fine-tuning is better and cheaper than in-context learning,” in Advances in Neural Information Processing Systems, eds. S. Koyejo, S. Mohamed, A. Agarwal, D. Belgrave, K. Cho, and A. Oh (Curran Associates, Inc.), 1950–1965.

[B11] Qi H. Dai Z. Huang C. (2025). Hybrid and unitary fine-tuning of large language models: Methods and benchmarking under resource constraints. arXiv preprint, arXiv:2507.18076.

[B12] Sanh V. Debut L. Chaumond J. Wolf T. (2019). Distilbert: a distilled version of bert: smaller, faster, cheaper and lighter. arXiv preprint, arXiv:1910.01108.

[B13] Sehanobish A. Dubey A. Choromanski K. Roy S. B. Jain D. Sindhwani V. . (2024). “Structured unrestricted-rank matrices for parameter efficient finetuning,” in Advances in Neural Information Processing Systems (Curran Associates, Inc.), 78244–78277. doi: 10.52202/079017-2487

[B14] Vaswani A. Shazeer N. Parmar N. Uszkoreit J. Jones L. Gomez A. N. . (2017). “Attention is all you need,” in Advances in Neural Information Processing Systems (Curran Associates, Inc.), 5998–6008.

[B15] Wang L. Chen S. Jiang L. Pan S. Cai R. Yang S. . (2025). Parameter-efficient fine-tuning in large language models: a survey of methodologies. Artif. Intell. Rev. 58:227. doi: 10.1007/s10462-025-11236-4

[B16] Wu Z. Arora A. Wang Z. Geiger A. Jurafsky D. Manning C. D. . (2024). “Reft: Representation finetuning for language models,” in Advances in Neural Information Processing Systems (Curran Associates, Inc.), 63908–63962. doi: 10.52202/079017-2041

[B17] Zhang D. Feng T. Xue L. Wang Y. Dong Y. Tang J. (2025). Parameter-efficient fine-tuning for foundation models. arXiv preprint, arXiv:2501.13787.

[B18] Zhang X. Zhao J. LeCun Y. (2015). “Character-level convolutional networks for text classification,” in Advances in Neural Information Processing Systems (Curran Associates, Inc.), 649–657.

